# Paediatric Physiotherapy curriculum: an audit and survey of Australian entry-level Physiotherapy programs

**DOI:** 10.1186/s12909-019-1540-z

**Published:** 2019-04-16

**Authors:** Karen Mistry, Emi Yonezawa, Nikki Milne

**Affiliations:** Faculty of Health Sciences and Medicine, Bond Institute of Health and Sport, Bond University, Robina, QLD, Gold Coast, 4226 Australia

**Keywords:** Australian, Physiotherapy, Paediatric, Curriculum, Entry-level, University, Tertiary education, Allied health, Training

## Abstract

**Background:**

No documented standard or core competencies exist for paediatric curriculum in entry-level physiotherapy programs in Australia. Consequently, extensive variability is thought to exist amongst Australian entry-level physiotherapy programs for preparing physiotherapists to work safely and effectively with children. The purpose of this study was to explore the landscape of paediatric curriculum in Australian entry-level physiotherapy programs and identify the paediatric content being covered, its perceived importance according to university academics who teach paediatrics, the mode of delivery and assessment, and the strengths, weaknesses, barriers and facilitators to implementing paediatric curriculum.

**Methods:**

A web-based desktop audit and an online cross-sectional survey using closed and open-ended questions was administered to all Australian universities offering entry-level physiotherapy programs in November 2017. Content coverage and perceived level of importance for paediatric content areas were determined using Likert scale responses. Open-ended responses were thematically analysed to identify key themes for strengths, weaknesses and facilitators to implementation of paediatric curriculum.

**Results:**

All (*n* = 20, 100%) entry-level programs used the terms lifespan, child and/or paediatrics somewhere in at least one subject descriptor. Forty-five percent (*n* = 9) of universities did not use the terms lifespan, child or paediatric in their published learning objectives. Eight (40%) universities offered a paediatric stand-alone course. Sixty-five (13/20) percent of universities invited, responded to the survey. For paediatric conditions the perceived level of importance was predominately higher than its course content coverage for 19 of the 31 conditions surveyed. Key barriers to implementating paediatric curriculum were: crowded curriculum, limited financial resources resulting in a lack of qualified staff, lack of prioritisation of paediatric curriculum and inadequate paediatric placement availability. Facilitators for effective implementation of paediatric content were stand-alone paediatric subjects, demonstrated dedication to paediatric curriculum and having suitably qualified faculty members.

**Conclusion:**

The results of this survey provide the physiotherapy community with the views of paediatric physiotherapy academic educators regarding the content, perceived need to expand content delivery in identified clinical areas, and the barriers and facilitators to implementing paediatric content in Australian entry-level physiotherapy programs. Further research exploring similar questions with paediatric physiotherapy clinicians would complement the findings of this study.

**Electronic supplementary material:**

The online version of this article (10.1186/s12909-019-1540-z) contains supplementary material, which is available to authorized users.

## Background

Physiotherapists in Australia received rights for being first-contact practitioners in 1978 [1]. These rights were accompanied with expectations to complete an entry-level degree in physiotherapy to be deemed capable of assessing and treating the general population throughout the lifespan. A guide for accreditation of entry-level physiotherapy programs published by the Australian Physiotherapy Council (APC) state that each university may design their own curriculum providing they meet the Australian and Aotearoa New Zealand Physiotherapy Practice Thresholds (2015) [2]. Additionally the APC state that Universities should do so while preparing students to be safe, effective and efficient entry-level physiotherapists [2]. Although physiotherapy programs in Australia prepare their own university-specific curriculum independently of each other, there is no standardised content that is expected or published for entry-level physiotherapy students to have covered in the field of paediatric physiotherapy. Paediatric physiotherapists are health professionals who work with children and adolescents and have a thorough understanding of child development and its relation to body systems and functions [3]. A paediatric physiotherapist should also follow a family-centred care approach that involves a decision-making process between families and professionals in a healthcare setting [4].

Approximately 19% of the Australian populations are under the age of 15 years [5]. In 2012, 4.2 million people in Australia were reported to have a disability, of which approximately 7% were children between the ages 0–17 years [6, 7]. Physiotherapy remains an area of increasing demand on the Australian occupational list in the medium to long term [8]. With the growing requirements of physiotherapists and increasing population of children, it is expected that most therapists will encounter children for therapy at some point in their career [9]. As Australia moves fully into the National Disability Insurance Scheme (NDIS) funded care for persons (including children) with a disability, it will likely benefit children and the physiotherapy profession to include and enhance paediatric curriculum in entry-level physiotherapy programs. The NDIS is a new model of care supporting people within the community who have a disability, their families and carers and focuses on a lifetime approach to enhance their future outcomes [10]. The skills and knowledge required to treat a child are not always transferrable from those learnt in adult contexts, as children with developmental difficulties and disabilities require specific care that is unique to their presentation and life context [9]. Whist all students in Australian physiotherapy programs will undertake clinical placements in adult care environments and will be assessed using a nationally standardised valid and reliable assessment tool for their clinical competencies [11–13], they may never undertake a clinical placement with the same level of external assessment in paediatric settings or with paediatric clients. Additionally, some students in Australia may graduate from their physiotherapy program having never been assessed for their competencies to safely and effectively assess and treat real infants or children, yet directly after graduating their program, they can become licenced to work independently with infants and children. For this reason, it may be appropriate to develop a minimum set of standards regarding paediatric-specific physiotherapy knowledge; skills and attributes which could be used to develop curriculum and a tool to assess paediatric competencies at entry-level to the profession, to better ensure that paediatric clients and their families are being effectively, efficiently and safely managed by physiotherapists across Australia.

### Review of literature

To understand issues surrounding paediatric content in physiotherapy programs globally, a narrative review of the literature was undertaken. Internationally, mixed method studies have been carried out between 1983 and 2017 utilising surveys, guidelines and opinions to determine paediatric physiotherapy curriculum standards. Fifteen studies investigating this topic have collectively involved 1700 participants; however, all studies were conducted in the United States (US) of America. Three key themes relating to paediatric physiotherapy curriculum have been identified in previously published literature including the following; (i) paediatric content (conditions and science); (ii) mode of delivery and; (iii) barriers to the implementation of paediatric curriculum. A review and synthesis of previously published literature suggests that only a minimal amount of paediatric content is covered within university curriculums in the US. Additionally, most US universities use a lifespan approach in the delivery of content, however, the time allocated to paediatric populations and approaches to care fluctuate substantially between programs [9, 14]. Discrepancies exist in the level of knowledge and skills of graduating physiotherapists from entry-level programs in the US. Insufficient faculty [15–19], insufficient clinical placements [14, 16] and a lack of consensus regarding paediatric content to prioritise [15, 16], were reasons provided in previously published literature, to explain the limited amount of paediatric content in US physiotherapy entry-level programs. In the development of first-contact practitioners who are licensed to work with infants and children, appropriate paediatric content delivered within the curriculum is critical to prepare physiotherapists to have adequate skills and knowledge to manage paediatric populations in a safe and effective manner. Considering the extensive variability in the paediatric content delivered across universities in the US, the Section on Paediatrics (SoP); a special interest group of APTA (American Physical Therapy Association) carried out an Education Summit in July 2012 [20]. An objective of this summit was to recommend strategies for academic institutions to assist in developing paediatric education to prepare entry-level physiotherapy students. Rapport, et al. [20] highlighted the decision-making process during the summit that resulted in a consensus on five core competencies that were referred to as essential knowledge base for all entry-level physiotherapy graduates regardless of the intent or interest to provide services to children upon completion of the program. The identified core competencies were: [1] Human development, [2] Age-appropriate patient/client management, [3] Family-centered care for all patient/client and family interactions, [4] Health promotion and safety, and [5] Legislative policy and systems. Additionally, the SoP, APTA [20] endorsed a list of paediatric conditions that are considered appropriate for inclusion in US entry-level physiotherapy programs.

Whilst paediatric core competencies exist in the US, there is currently no documented standard for university curriculum or competencies relevant to paediatric physiotherapy in Australia for which the Australian Physiotherapy Council (APC) can hold programs accountable. While the APC commonly reviews subject outlines and descriptors as well as assessment items during accreditation cycles [2], historically there has not been a consistent requirement to detail the paediatric curriculum of a program. This is despite there being a common acknowledgement of very limited paediatric placement experiences where external assessment is available. Subsequently with a lack of available evidence, the accreditation panels may be certifying universities to graduate first-contact, entry-level physiotherapists who may not have the skills or knowledge to work safely and effectively with children yet, can become registered to do so. Now that the NDIS has been implemented across Australia it is a critical point in time where Australian physiotherapy program accreditation panels could review their expectations regarding paediatric curriculum, including content, delivery and achieved competencies. To become a NDIS provider, registration can be completed online following a checklist application process ensuring providers understand this scheme and its role within the community. Providers with the intention to treat children aged between zero - six years have to register as an Early Childhood Early Intervention (ECEI) provider through the NDIS. To become an ECEI provider evidence of previous engagement with children (0–6 years) is required for registration. However, approval to become a standard NDIS provider to treat children over the age of seven years, requires no verified proof of paediatric experience, knowledge or competency [21]. Furthermore, private practice physiotherapists are not required to provide evidence of competencies to work with children of any age or ability. Therefore, a registered physiotherapist could potentially be providing care to children within the community without any history of previously assessing or treating real-life children in a safe, effective and efficient manner and this is also an area worthy of further consideration at an accreditation level.

The exploration and documentation of current paediatric curriculum in entry-level physiotherapy programs in Australia, may be a useful mapping process for universities as a bench-marking opportunity. This process may assist universities to become more accountable for developing in students, the knowledge and skills required of entry-level, first-contact practitioners to practice with children and infants. Therefore, the objective of this prospective cross-sectional study was to investigate the landscape of paediatric physiotherapy curriculum in entry-level physiotherapy programs in Australia using two methodological steps. The first preliminary step was to undertake a desktop-audit, with the aim of quantifying publicly available information (e.g. subject outlines / course descriptors) regarding paediatric-specific learning objectives and assessment items published on university websites for entry-level physiotherapy programs. Secondly, the research team undertook a national survey with the aim of identifying:

(i) The paediatric curriculum content covered in entry-level physiotherapy programs and to what extent;
(ii) The perceived importance of paediatric content by university academics who teach or convene courses inclusive of paediatric content in entry-level programs, including differences in responses based on program level (i.e. bachelor versus entry level masters +/− extended);
(iii) The mode of delivery of paediatric curriculum and assessment in entry-level programs;
(iv) Strengths, weaknesses, barriers and facilitators, to the implementation of paediatric coursework curriculum in entry-level programs.

## Methods

### Participants

Details for all universities offering entry-level physiotherapy programs throughout Australia (*n* = 20 universities) were sourced using the Australian Health Practitioner Regulation Agency (AHPRA) website [22]. All Australian entry-level physiotherapy programs were eligible to be included in this study. A list of names, emails and phone numbers for staff members teaching directly into the physiotherapy curriculum at respective universities were collected using publicly accessible university websites. Twenty universities were identified and from these universities, thirty-one possible participants were invited to participate in the research study, with the expectation of having one person per university program participate in the study to share information about their university curriculum.

### Research design (desktop audit and survey)

A web-based desktop audit of university entry-level curriculum and a mixed method cross-sectional survey was concurrently carried out to review each university’s (*n* = 20) curriculum for paediatric content as it was published on their university website at the time of the audit. The inclusion criteria for both research designs were as follows: [1] University or Institution of higher education located in Australia, [2] Offering an entry-level physiotherapy program (i.e. bachelor, masters by coursework, extended masters). Institutions offering only a diploma or certificate of trade in the area of physiotherapy were excluded. The desktop audit captured the program type (i.e. bachelor, masters by coursework, extended masters) for each university, and included the course description, learning outcomes, syllabus and assessments for each subject that included the terms child, paediatric or lifespan, if available. The desktop audits were categorised into three groups for analysis: i) all universities, ii) survey respondents and; iii) non-survey respondents. Upon reviewing information about each course as it was published on the web, four main questions were answered to explore paediatric content covered in universities. Responses to questions were scored 0 or 1 (0 = No and 1 = Yes). The questions addressed in the desk-top audit were: (i) are any of the terms - child, lifespan or paediatric used in the published curriculum? (ii) did the course description use terms such as child, lifespan or paediatric but did not use these terms in the learning objectives? (iii) was there clearly documented paediatric specific assessment in the curriculum? and (iv) was a stand-alone paediatric course/subject offered?

A mixed method cross-sectional study including quantitative and qualitative methods was used to collect data. Ethical approval was obtained from the Human Research Ethics committee at the host University (HREC approval number: 16162). An online cross-sectional survey was developed in three stages; (i) an extensive review of existing literature assisted the research team in drafting the survey; (ii) local paediatric physiotherapists (*n* = 3) with higher education teaching experience were consulted to pilot the survey and provide feedback on the content, themes, structure and assist with identifying any question ambiguity; (iii) the survey was modified based on feedback before circulation of the survey link to potential study participants using Survey Monkey. The final survey design consisted of six themes including the following; (i) paediatric teaching staff demographic; (ii) curriculum relating to (ii.a.) knowledge of typical development; (ii.b.) paediatric diagnoses (atypical development); (iii) paediatric examination/assessment; (iv) paediatric intervention and; (v) strengths, weaknesses, barriers and facilitators to implementation. A five-point Likert scale was used to quantify the content covered in the university’s curriculum at the time of the survey (0 = not covered at all, 5 = covered very well) and the teaching staffs’ perceived importance in covering that content (0 = strongly disagree with its importance, 5 = strongly agree with its importance). Questions using dichotomous answers (Yes / No) were employed to explore barriers to implementing paediatric curriculum. Open-ended questions were employed to identify facilitators to implementing paediatric curriculum within entry-level physiotherapy programs and to explore strengths and weakness’ in the paediatric curriculum. (See Additional file 1 for copy of survey).

### Procedure

The chief investigator sent an e-mail invitation to all persons (*n* = 31) identified as suitable to participate in the study including a link to Survey Monkey where the participant information sheet and consent form were accessible. After providing consent, participants were asked to complete the online survey (taking approximately 30 min). A fortnightly e-mail was sent out as a reminder to participate in the study. On completion of the survey, the results of the university desktop audit were emailed to the relevant universities. To validate the responses from the survey when ambiguous answers were provided, participants were provided an opportunity to update the details from the desktop audit for their university’s paediatric curriculum. This step was undertaken by sending a Word document file, (containing the extracted paediatric content from the university website) via email to the participants at the relevant universities.

### Analysis of data

Survey Monkey data was retrieved as an Excel file and coded before importing into SPSS version 24 [23] for analysis and storage. Descriptive statistics were used to derive percentages and frequencies for demographic information, barriers to the implementation of paediatric curriculum and results from the desktop audit. Median and mean responses with standard deviations were calculated for all questions using a Likert scale. Tests for normality were performed and assumptions explored to identify the appropriate analyses to undertake. After ensuring that all relevant assumptions were met, Mann-Whitney U tests were undertaken as a secondary analysis to identify if significant differences in median results existed between bachelor level programs and entry-level master programs (+/− extended) for content delivered and perceived importance of content in programs. A *p*-value of < 0.05 was considered significant. To examine the impact of non-paediatric therapist responses to the median scores, a sensitivity analysis was undertaken where the responses of non-paediatric physiotherapists (*n* = 2) were removed from the raw data to identify potential differences in median values compared to initial analyses. Two forms of thematic analysis were applied to open-ended questions. Open coding was used to identify themes for strengths and weaknesses of the curriculum concerning paediatric content [24]. Surface analysis [24] of frequently used words were utilised to identify themes for facilitators to the implementation of paediatric curriculum and inter-professional education [24].

## Results

### Paediatric teaching staff demographic

From the 31 possible participants from 20 Australian universities with entry-level physiotherapy programs, 16 physiotherapists who were involved with paediatric curriculum across Australian universities initially agreed to participate (response rate 65%). Of the survey responders eight bachelor and six master (+/− extended) programs were represented in this study. One person withdrew their consent after identifying that they did not have the required paediatric curriculum experience to complete the survey. Among the remaining 15 participants, most were females (*n* = 14) with an average of six years of teaching experience (ranging from 1 to 12 years) and an average of 22 years of clinical experience (ranging from 9 to 38 years) as a registered physiotherapist. Eighty-seven percent (*n* = 13) of survey respondents undertook their physiotherapy entry-level training in Australia with the remaining participants completing their physiotherapy training overseas (*n* = 2, 13.3%). The highest level of academic degree completed by participants was a PhD (*n* = 7, 46.7%), with others completing degrees in, bachelor +/− honours (*n* = 3, 20%) and master level degrees (*n* = 6, 40.0%). Additionally, some participants had completed post-graduate training in a variety of fields including paediatrics (*n* = 7, 46.7%), education (*n* = 4, 26.7%), cardiopulmonary (*n* = 3, 20.0%) and neurodevelopmental therapy (*n* = 2, 13.3%). Some participants also completed additional training in other fields including musculoskeletal (*n* = 1, 6.7%), sports (*n* = 1, 6.7%), rural and remote physiotherapy (*n* = 1, 6.7%), aquatic physiotherapy (*n* = 1, 6.7%) and leadership (*n* = 1, 6.7%). Sixty-seven percent of participants (*n* = 10) were in a part-time position averaging 0.56 full-time equivalent (FTE) (ranging from 0.4 to 0.8 FTE). The other 33% (*n* = 5) of the participants were employed in a full-time position as a paediatric academic staff member. Ten participants (66.7%) were employed at an academic level of Lecturer, four as Senior Lecturer (26.7%) or equivalent and one respondent was employed at the Associate Professor level (6.7%).

### Desktop audit of Paediatric content in entry-level Physiotherapy programs

Publicly available information was recorded and synthesized for each entry-level physiotherapy program in Australia. All (*n* = 20, 100%) entry-level programs at the time of this study were noted to use the terms ‘lifespan’ and/or ‘child’ and/or ‘paediatrics’ somewhere in at least one of their subject descriptors. Forty-five percent (*n* = 9) of the universities did not use the terms ‘lifespan, child or paediatric’ in their published learning objectives and four of these universities did not respond to the survey invitation. Fourteen (70%) universities either did not specify or did not make publicly available items of assessment for paediatric specific learning. Eight (40%) universities offered a paediatric stand-alone course and six of these universities were survey responders.

### Survey responses for knowledge of typical development

Table 1, outlines response rates relevant to content covered in programs and the perceived importance of the content areas relevant to typical development. Most content areas were reported to be ‘somewhat’ to ‘very well’ covered except for ‘knowledge of prenatal development and birth’, ‘when a child should provide consent’ and ‘milestones in social-emotional, speech and language domains’ where the majority of responses were ‘not very well’ or ‘not at all’ covered in the curriculum. Most of the responses for questions relating to the perceived importance of knowledge relevant to typical development were ‘agreed’ to ‘strongly agreed’. There was one ‘disagree’ response for ‘develop foundation knowledge for prenatal development and birth’ and four ‘neutral’ responses, including ‘childhood development and learning’ (*n* = 1), developmental motor milestones (*n* = 1) and milestones in social-emotional, speech and language domains (*n* = 2).

**Table 1:** Content covered and perceived importance reported for the knowledge of typical development

*n* = 15	Content covered							Perceived Importance						
	0 Not at all (%)	1 Not very well (%)	2 Some-what (%)	3 Well (%)	4 Very-well (%)	Median	Mean (SD)	0 Strongly disagree (%)	1 Disagree (%)	2 Neutral (%)	3 Agree (%)	4 Strongly agree (%)	Median	Mean (SD)
Develop foundation knowledge of prenatal development and birth	1 (6.7)	1 (6.7)	3 (20.0)	5 (33.3)	5 (33.3)	3 (well)	2.80 (1.21)	–	1 (6.7)	–	8 (53.3)	6 (40.0)	3 (agree)	3.3 (0.80)
Develop foundation knowledge of the theories of childhood development and learning	–	–	4 (26.7)	4 (26.7)	7 (46.7)	3 (well)	3.20 (0.86)	–	–	1 (6.7)	5 (33.3)	9 (60.0)	4 (strongly agree)	3.5 (0.64)
Demonstrate knowledge of developmental motor milestones	–	–	2 (13.3)	4 (26.7)	9 (60.0)	4 (very well)	3.5 (0.74)	–	–	1 (6.7)	2 (13.3)	12 (80.0)	4 (strongly agree)	3.7 (0.59)
Understand the importance of therapeutic play within diverse family, cultural, community and societal context	–	–	1 (6.7)	6 (40.0)	8 (53.3)	4 (very well)	3.5 (0.64)	–	–	–	2 (13.3)	13 (86.7)	4 (strongly agree)	3.9 (0.35)
Understand when a child should provide consent and gaining parent/ carer consent	–	1 (6.7)	1 (6.7)	6 (40.0)	7 (46.7)	3 (well)	3.3 (0.88)	–	–	–	2 (13.3)	13 (86.7)	4 (strongly agree)	3.9 (0.35)
Demonstrate knowledge of developmental milestones in the social-emotional, speech and language domains	–	1 (6.7)	5 (33.3%)	6 (40.0)	3 (20.0)	3 (well)	2.7 (0.88)	–	–	2 (13.3)	3 (20.0)	10 (66.7)	4 (strongly agree)	3.5 (0.74)

*SD* Standard Deviation

### Survey responses for paediatric diagnosis (atypical development)

**Fig. 1:**
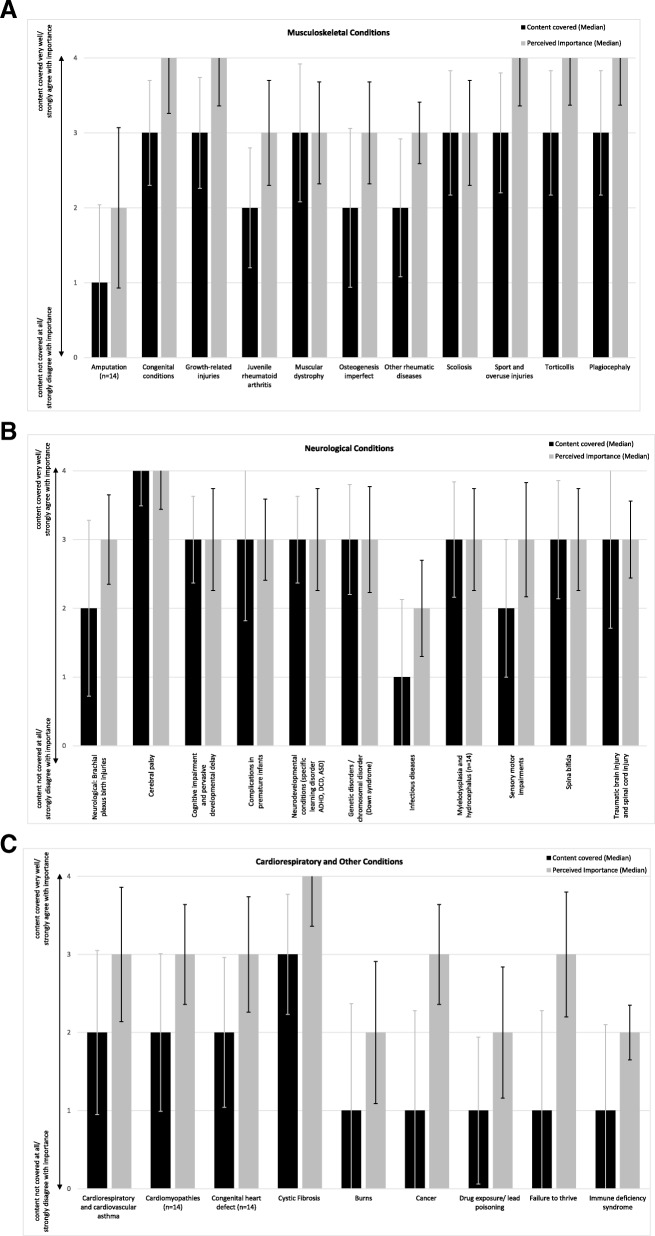
a. Median responses to questions about content covered and perceived importance for musculoskeletal conditions. b. Median responses to questions about content covered and perceived importance for neurological conditions. c. Median responses to questions about content covered and perceived importance for cardiorespiratory and other conditions

Fig. 1 (a, b and c) illustrates the median responses (content covered and perceived importance) for questions about paediatric specific diagnoses. Perceived importance responses were equal to or more than one value higher on the 5-point Likert scale than responses to content covered for 19 of the 31 conditions surveyed. Responses for ‘cancer’ and ‘failure to thrive’ revealed that the median response for content covered was in the ‘not very well’ range [1] but the median response for perceived importance was ‘agree’ [3], therefore revealing a 2-point difference on a five-point scale. Content coverage and perceived importance values were equal for 10 of the surveyed conditions. Mann-Whitney U tests revealed no significant differences between bachelor and master level programs for content coverage except for neurodevelopmental conditions covered (e.g. specific learning disorder, ADHD – Attention Deficit Hyperactivity Disorder, DCD – Developmental Coordination Disorder, ASD – Autism Spectrum Disorder) which were reported to be covered in more detail in bachelor programs (Mdn bachelor = 3.0, Mdn master = 2.0, U = 10.00, *p* = 0.025) and Spina Bifida which was reported to be covered more in master level programs (Mdn bachelor = 3.0, Mdn master = 4.0, U = 6.5, *p* = 0.008). For the perceived importance of curriculum areas, there were no significant differences between bachelor and master level programs, other than for growth-related injuries (Mdn bachelor = 3.0, Mdn master = 4.0, U = 9.00, *p* = 0.014) and sports and overuse injuries (Mdn bachelor = 3.0, Mdn master = 4.0, U = 6.0, *p* = 0.005) where in both cases, the master level programs felt they were more important. When two responders who were not paediatric physiotherapists were removed in the sensitivity analysis the median perceived importance in paediatric curriculum for ‘cardiomyopathies’, ‘traumatic brain and spinal cord injury’, ‘cancer’ and ‘failure to thrive’ decreased from ‘agree’ [3] to ‘neutral’ [2] for each condition. Additionally, the significant difference between the bachelor and master level programs for content covered relevant to neurodevelopmental conditions was no longer apparent (Mdn bachelor = 3; Mdn master = 2, U = 8.50, *p* = 0.06). However, the significant differences noted for the perceived importance of covering growth-related injuries (Mdn bachelor = 3, Mdn master = 4, U = 7.50, *p* = 0.034) and sports and overuse injuries (Mdn bachelor = 3, Mdn master 4, U = 5.0, *p* = 0.014) in the curriculum remained significantly different between bachelor and master level programs.

### Survey responses for mode of delivery and assessment of paediatric learning

Only five (33.3%) responders reported that they did not use written exams or quizzes to assess paediatric learning outcomes. Practical exams (Objective Structural Clinical Examination (OSCE) or Viva) and seminar/oral presentations were used by 62.5 and 53.3% of responders, respectively. Seven (46.7%) of responders stated that clinical placements were used to assess paediatric learning outcomes for all students in their program. Eighty-seven percent (*n* = 13) of participants reported that paediatric content was provided to students through lectures and tutorials. Problem/case-based learning (*n* = 10, 66.7%), independent study (*n* = 10, 66.7%), online content/modules (*n* = 10, 66.7%) and clinical placements (*n* = 10, 66.7%) were other popular modes of delivery for paediatric curriculum. Some programs also reported using workshop/practical classes (*n* = 9, 60%), simulated learning (*n* = 9, 60%), flip classes (*n* = 4, 27.7%) and site visits (*n* = 3, 20.0%) in the delivery of paediatric content. Eleven (73.0%) of the 15 responders indicated that paediatric curriculum in their program was delivered across different subjects as a lifespan approach and nine (60%) responders stated that their program had a stand-alone paediatric subject.

### Survey responses for paediatric examination/assessment

Table 2 outlines the responses relevant to paediatric assessment and examination. The data revealed that all content was covered ‘somewhat’ (score = 2) to ‘very well’ (score = 4) with the exception of ‘ergonomic and body mechanics’, ‘orthotic, protective and supportive devices’, ‘reflex integrity’, ‘ventilation and respiration / gas-exchange’ and ‘cardiorespiratory fitness’ which were covered ‘not very well’ (score = 1) or ‘not at all’ (score = 0). Most responses regarding the perceived importance of assessment/examinations were ‘agree’ (score = 3) to ‘strongly agree’ (score = 4) except for ‘ergonomics and body mechanics’, ‘motor skills’, ‘neuromotor and sensory assessment’, ‘orthotic, protective and supportive devices’, ‘range of motion’ and ‘ventilation and respiration / gas-exchange’ which received ‘neutral’ (score = 2) responses. Mann-Whitney U tests revealed no significant differences between bachelor and masters level responses. After removing the non-paediatric physiotherapists in the sensitivity analysis, the median value for content covered for ‘reflex integrity’ reduced from 3 (well) to 2 (somewhat) and for ‘outcome measures’ increased from 3 (well) to 4 (very well). Additionally, the sensitivity analysis did not reveal any significant differences between bachelor and master level programs for content covered and perceived importance of paediatric examination / assessment.

**Table 2:** Content covered and perceived importance reported for paediatric examination/assessment

*n* = 15	Content covered							Perceived Importance						
	0 not at all	1 not very well	2 somewhat	3 well	4 very well	Median	Mean (SD)	0 strongly disagree	1 disagree	2 neutral	3 agree	4 strongly agree	Median	Mean (SD)
Client/parent interview	–	–	1 (6.7%)	5 (33.3%)	9 (60.0%)	4 (very well)	3.5 (0.64)	–	–	–	1 (6.7%)	14 (93.3%)	4 (strongly agree)	3.9 (0.26)
Physical assessment	–	–	1 (6.7%)	6 (40.0%)	8 (53.3%)	4 (very well)	3.5 (0.64)	–	–	–	2 (13.3%)	13 (86.7%)	4 (strongly agree)	3.9 (0.45)
Ergonomics and body mechanics	–	1 (6.7%)	9 (60.0%)	2 (13.3%)	3 (20.0%)	2 (somewhat)	2.5 (0.92)	–	–	2 (13.3%)	7 (46.7%)	6 (40.0%)	3 (agree)	3.3 (0.70)
Gait, locomotion and balance	–	–	4 (26.7%)	6 (40.0%)	5 (33.3%)	3 (well)	3.1 (0.80)	–	–	–	4 (26.7%)	11 (73.3%)	4 (strongly agree)	3.7 (0.46)
Joint integrity and mobility	–	–	4 (26.7%)	8 (53.3%)	3 (20.0%)	3 (well)	2.9 (0.70)	–	–	–	9 (60.0%)	6 (40.0%)	3 (agree)	3.4 (0.51)
Motor control and motor learning	–	–	4 (26.7%)	5 (33.3%)	6 (40.0%)	3 (well)	3.1 (0.83)	–	–	–	4 (26.7%)	11 (73.3%)	4 (strongly agree)	3.7 (0.46)
Motor skills	–	–	1 (6.7%)	5 (33.3%)	9 (60.0%)	4 (very well)	3.5 (0.64)	–	–	1 (6.7%)	2 (13.3%)	12 (80.0%)	4 (strongly agree)	3.7 (0.59)
Muscle performance (strength, power, endurance)	–	–	1 (6.7%)	11 (73.3%)	3 (20.0%)	3 (well)	3.1 (0.52)	–	–	–	7 (46.7%)	8 (53.3%)	4 (strongly agree)	3.5 (0.52)
Neuromotor and sensory assessment (development and integration)	–	–	5 (33.3%)	4 (26.7%)	6 (40.0%)	3 (well)	3.1 (0.88)	–	–	1 (6.7%)	6 (40.0%)	8 (53.3%)	4 (strongly agree)	3.5 (0.64)
Orthotic, protective and supportive devices	1 (6.7%)	2 (13.3%)	8 (53.3%)	4 (26.7%)	–	2 (somewhat)	2 (0.85)	–	–	2 (13.3%)	8 (53.3%)	5 (33.3%)	3 (agree)	3.2 (0.68)
Posture	–	–	4 (26.7%)	7 (46.7%)	4 (26.7%)	3 (well)	3 (0.76)	–	–	–	9 (60.0%)	6 (40.0%)	3 (agree)	3.4 (0.51)
Range of motion (muscle length)	–	–	1 (6.7%)	10 (66.7%)	4 (26.7%)	3 (well)	3.2 (0.56)	–	–	1 (6.7%)	8 (53.3%)	6 (40.0%)	3 (agree)	3.3 (0.62)
Reflex integrity	–	1 (6.7%)	6 (40.0%)	6 (40.0%)	2 (13.3%)	3 (well)	2.6 (0.83)	–	1 (6.7%)	2 (13.3%)	7 (46.7%)	5 (33.3%)	3 (agree)	3.1 (0.88)
Ventilation and respiration/ gas exchange	–	1 (6.7%)	3 (20.0%)	8 (53.3%)	3 (20.0%)	3 (well)	2.9 (0.83)	–	–	2 (13.3%)	7 (46.7%)	6 (40.0%)	3 (agree)	3.3 (0.70)
Cardiorespiratory fitness	–	1 (6.7%)	3 (20.0%)	8 (53.3%)	3 (20.0%)	3 (well)	2.9 (0.83)	–	–	–	8 (53.3%)	7 (46.7%)	3 (agree)	3.5 (0.52)
Outcome measures (AIMS, NSMDA, GMFCS, BOT-2, MABC 2, TGMD2)	–	–	1 (6.7%)	7 (46.7%)	7 (46.7%)	3 (well)	3.4 (0.63)	–	–	–	5 (33.3%)	10 (66.7%)	4 (strongly agree)	3.7 (0.49)
Clinical reasoning tools (ICF)	–	–	–	2 (13.3%)	13 (86.7%)	4 (very well)	3.9 (0.35)	–	–	–	2 (13.3%)	13 (86.7%)	4 (strongly agree)	3.9 (0.35)
Understanding the role of the members of the interprofessional paediatric team	–	–	–	6 (40.0%)	9 (60.0%)	4 (very well)	3.6 (0.51)	–	–	–	3 (20.0%)	12 (80.0%)	4 (strongly agree)	3.8 (0.41)

*SD* Standard deviation, *AIMS* Alberta Infant Motor Scale, *NSMDA* Neurosensory Motor Developmental Assessment, *GMFCS* Gross Motor Function Classification Scale, *BOT-2* Bruininks-Oseretsky Test of Motor Proficiency 2nd Edition, *MABC 2* Movement ABC 2nd Edition, *TGMD2* Test of Gross Motor Development 2nd Edition, *ICF* International Classification of Function Disability and Health

### Survey responses for paediatric interventions

Table 3 outlines the responses related to content coverage and perceived importance of paediatric interventions. More than half of surveyed paediatric interventions resulted in equal median scores when comparing curriculum content coverage (‘well’ to ‘very well’) and its perceived importance (‘agree’ to ‘strongly agree’). Five intervention types were perceived as more important than what was currently being covered in the curriculum (based on perceived important median scores being one point higher than the median score for the same content area coverage). Electrotherapeutic and mechanical modalities as well as behaviour management strategies were reported to be only ‘somewhat’ [2] covered in paediatric curriculum and electrotherapeutic and mechanical modalities were perceived to be only ‘somewhat’ [2] important to cover. Mann-Whitney U tests revealed no significant differences between responses for bachelor and masters level programs for survey responses to content covered or perceived importance. After removing non-paediatric physiotherapists from the dataset in the sensitivity analysis the perceived importance of ‘therapeutic exercises’ increased from 3 (agree) to 4 (strongly agree) and the content covered for ‘prescription and application of equipment devices’ reduced from 3 (well) to 2 (somewhat). The sensitivity analysis did not reveal any significant differences between bachelor and master level programs for content covered and perceived importance of any paediatric interventions.

**Table 3:** Content covered and perceived importance reported for paediatric interventions

*n* = 15	Content covered							Perceived Importance						
	0 not at all	1 not very well	2 Some-what	3 well	4 very well	Median	Mean (SD)	0 strongly disagree	1 disagree	2 neutral	3 agree	4 strongly agree	Median	Mean (SD)
Manual therapy or positioning and handling	–	1 (6.7%)	4 (26.7%)	7 (46.7%)	3 (20.0%)	3 (well)	2.8 (0.86)	–	–	–	10 (66.7%)	5 (33.3%)	3 (agree)	3.3 (0.49)
Family/ patient-centered care	–	–	–	4 (26.7%)	11 (73.3%)	4 (very well)	3.7 (0.46)	–	–	–	2 (13.3%)	13 (86.7%)	4 (strongly agree)	3.9 (0.35)
Therapeutic exercises	–	1 (6.7%)	2 (13.3%)	10 (66.7%)	2 (13.3%)	3 (well)	2.9 (0.74)	–	–	2 (13.3%)	6 (40.0%)	7 (46.7%)	3 (agree)	3.3 (0.72)
Play-based exercises	–	–	1 (6.7%)	9 (60.0%)	5 (33.3%)	3 (well)	3.3 (0.59)	–	–	–	3 (20.0%)	12 (80.0%)	4 (strongly agree)	3.8 (0.41)
Functional training in self-care and in-home management	–	–	5 (33.3%)	6 (40.0%)	4 (26.7%)	3 (well)	2.9 (0.80)	–	–	1 (6.7%)	6 (40.0%)	8 (53.3%)	4 (strongly agree)	3.5 (0.64)
Functional training for use in school or play, in the community and leisure integration/ re-integration	–	–	4 (26.7%)	6 (40.0%)	5 (33.3%)	3 (well)	3.1 (0.80)	–	–	1 (6.7%)	5 (33.3%)	9 (60.0%)	4 (strongly agree)	3.5 (0.64)
Prescription and application of equipment and devices (assistive devices, orthotics, prosthetics)	–	3 (20.0%)	4 (26.7%)	8 (53.3%)	–	3 (well)	2.3 (0.82)	–	1 (6.7%)	2 (13.3%)	8 (53.3%)	4 (26.7%)	3 (agree)	3.0 (0.85)
Airway clearance techniques	–	–	2 (13.3%)	10 (66.7%)	3 (20.0%)	3 (well)	3.1 (0.59)	–	–	2 (13.3%)	8 (53.3%)	5 (33.3%)	3 (agree)	3.2 (0.68)
Electrotherapeutic and mechanical modalities	4 (26.7%)	2 (13.3%)	6 (40.0%)	3 (20.0%)	–	2 (somewhat)	1.5 (1.13)	2 (13.3%)	2 (13.3%)	8 (53.3%)	3 (20.0%)	–	2 (somewhat)	1.8 (0.94)
Behaviour management	2 (13.3%)	3 (20.0%)	6 (40.0%)	3 (20.0%)	1 (6.7%)	2 (somewhat)	1.9 (1.13)	–	–	3 (20.0%)	11 (73.3%)	1 (6.7%)	3 (agree)	2.9 (0.52)
Understanding the role of the members of the interprofessional paediatric team	–	–	3 (20.0%)	6 (40.0%)	6 (40.0%)	3 (well)	3.2 (0.77)	–	–	–	4 (26.7%)	11 (73.3%)	4 (strongly agree)	3.7 (0.46)
Use of goal setting for treatment planning	–	–		5 (33.3%)	10 (66.7%)	4 (very well)	3.7 (0.49)	–	–	–	3 (20.0%)	12 (80.0%)	4 (strongly agree)	3.8 (0.41)

*SD* Standard Deviation

### Survey responses for paediatric curriculum strengths, weaknesses, facilitators and barriers

**Fig. 2:**
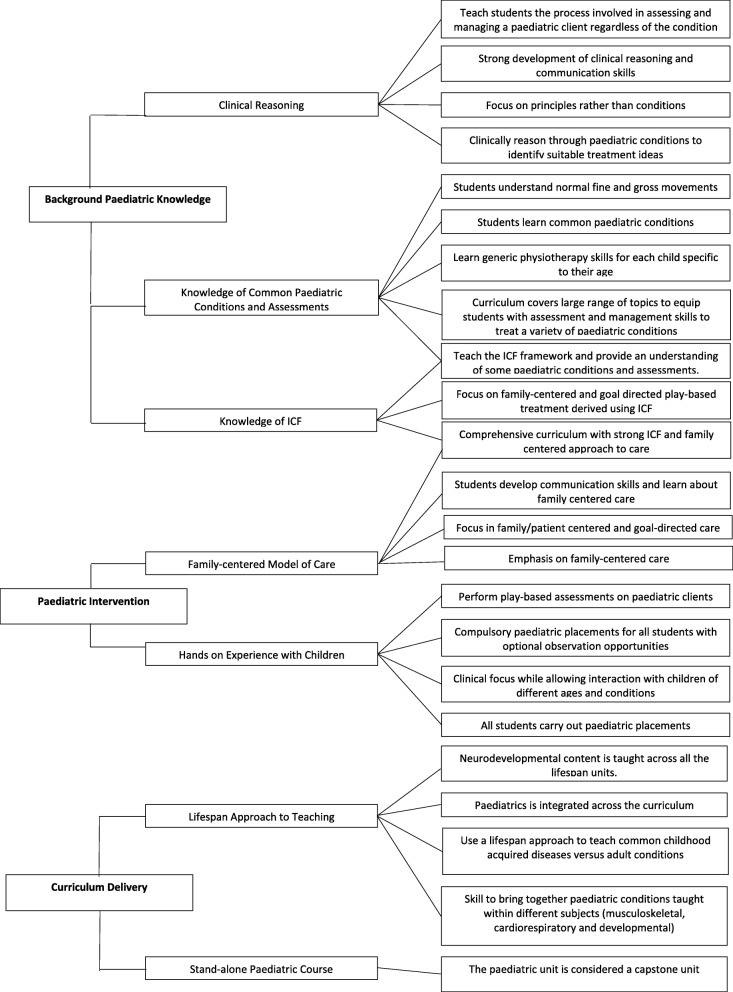
Thematic analysis for open-ended responses related to strengths of curriculum covering paediatric content

**Fig. 3:**
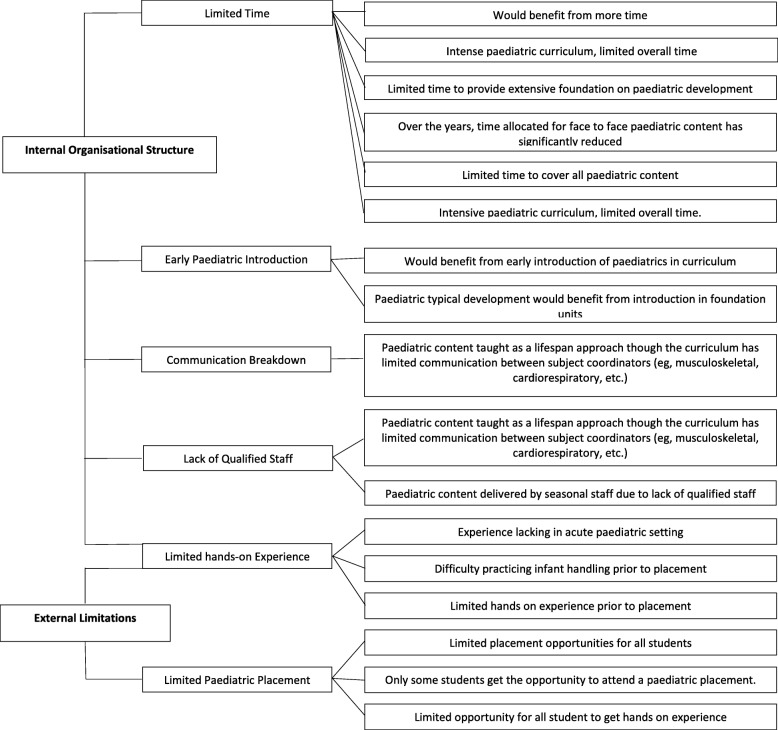
Thematic analysis for open-ended responses related to weaknesses of curriculum covering paediatric content

Figs. 2 and 3 outline the thematically analysed responses (*n* = 15) to open-ended survey questions regarding strengths and weaknesses of curriculum with paediatric content. Three key themes relevant to paediatric curriculum strengths emerged from this thematic analysis including: i) background paediatric/development knowledge; ii) paediatric treatment skills and; iii) mode of curriculum delivery. Sixty-seven percent of participants (*n* = 10) highlighted one of their greatest paediatric curriculum strengths to be teaching their students common paediatric conditions along with clinical reasoning skills that equip students to treat a wide paediatric caseload (see Fig. 2). The thematic analysis of weaknesses relevant to paediatric curriculum revealed two key themes: i) internal organisation structural limitations and ii) external limitations (see Fig. 3). Forty percent of participants (*n* = 6) identified ‘limited time allocated’ to learning paediatric skills and content, as the greatest weakness of the paediatric curriculum, which was themed under internal organisation structural limitations. The external limitations highlighted by 40% of the participants (*n* = 6) were ‘inadequate placement availabilities’, resulting in students having limited hands on experience with the paediatric population (see Fig. 3).

Surface thematic analysis of participants’ most frequently reported responses to open-ended questions regarding ‘facilitators to implementation and development of paediatric curriculum’ revealed three key themes which are outlined below with example responses supporting the themes:

(i) Stand-alone course

*“would benefit from embedding the main paediatric content within one subject instead of it being spread out over 4 years.”*

(ii) Demonstrated dedication to implementation of a paediatric curriculum

*“staff members covering paediatric subjects are very passionate about emphasising the paediatric component in the course and making it as relevant and engaging as possible for students.”*

*“supportive program director and strong representation of paediatric staff”*

(iii) Having suitably qualified faculty members.
*“strong clinicians from a variety of paediatric speciality areas are involved in both curriculum development and teaching.”*

Two major themes were identified through thematic analysis of open-ended questions regarding ‘facilitators of inter-professional education and training implementation in paediatrics’:

(i) dedication to implement a multidisciplinary team approach in teaching

*“key staff that understand the importance of interprofessional education are dedicated to see more development within programs.”*

(ii) maintaining relationships with multidisciplinary teams (MDTs) within the university and external partners.

*“Have good links and a number of subjects that are run interprofessionally with the occupational therapy and speech pathology department at our university.”*

Crowded curriculum (*n* = 12, 80.0%) was identified as a primary barrier to the implementation and development of paediatric physiotherapy curriculum. Limited financial resources (*n* = 6, 40.0%) and lack of prioritisation of curriculum space for paediatric content (*n* = 6, 40.0%) were also identified as barriers. Regarding the implementation and development of Interprofessional health Education (IPE) and training for students in the field of child health and development the following barriers were identified; (i) timetabling challenges (*n* = 14, 93.3%), (ii) crowded curriculum (*n* = 10, 66.7%) and; (iii) organisational structure of the institution of higher education (*n* = 9, 60.0%).

Additional comments regarding paediatric curriculum in entry-level programs were offered by seven participants and two responses that were otherwise not represented in previous results are provided below.

*“I think now is a crucial time to push the importance of minimum level standards upon graduation, given the introduction of NDIS.”*

*“Always need to stretch the creativity on how to assess entry-level practitioners in a standardised way for safety and efficacy to practice in paediatrics. Basic practice standards really need to be updated and finalised by the APA, so curriculums can align to these.”*

## Discussion

### Major findings

This study is the first to investigate the paediatric physiotherapy curriculum in entry-level physiotherapy programs in Australia. In relation to Australian physiotherapy entry-level programs, the preliminary step undertaken was a desktop-audit, with the aim of quantifying the publicly available information regarding paediatric-specific learning objectives and assessment items published on the university website. Through a national survey, this study identified; (i) paediatric curriculum content covered and to what extent; ii) the perceived importance of academic content by university academics teaching or convening subjects inclusive of paediatric content, including differences in responses based on program level (i.e. bachelor versus entry level masters +/− extended); iii) the mode of delivery of paediatric curriculum and assessment; (iv) barriers and/or facilitators to the implementation of paediatric coursework. The major findings of this study suggest that most paediatric content outlined in the present survey was reported as being covered ‘well’ in Australian universities with very few topics being covered ‘very well’. Additionally, the perceived importance for paediatric content coverage was greater for most topics surveyed when compared to the level of actual coverage reported in the programs. Whilst curriculum delivery and assessment modes reported in the survey differed across universities, the desktop audit revealed that just under half of Australian universities did not use the terms lifespan or paediatric in their published learning outcomes, suggesting that learning specific to paediatric caseloads were not targeted outcomes. Limited time allocated to paediatric content was the most commonly reported weakness of paediatric curriculum with crowded curriculum being identified as the primary barrier to the implementation/delivery of paediatric content. Further, the most commonly reported facilitators to the effective implementation of paediatric curriculum was having a stand-alone paediatric subject and having both champions for implementation of paediatric content and suitably qualified physiotherapists on staff to teach the content. Whilst these themes clearly arose from the survey analysis, further work is required to assist with determining how much time should be dedicated to paediatrics and what modes of delivery best meet the needs of new graduate physiotherapists to safely and effectively service paediatric clients. The US based education summit carried out in 2012 by SoP, APTA [20] discussed the number of hours to be devoted to teaching paediatrics but were unable to reach a consensus due to the lack of available evidence at the time. The SoP, APTA determined that each educational program could continue making their own decisions about hours and mode of delivery as so long as a variety of learning strategies were used. With relation to mode of delivery the experts attending the SoP, APTA summit highlighted the two most common approaches for content delivery were integrated or a stand-alone approach. The integrated approach would require the education programs to deliver paediatric content across the curriculum by providing foundation knowledge relevant to paediatrics in the first year of study and as the years progress to build on this knowledge through the different topics in the physiotherapy curriculum. A unique challenge identified with the integrated program was the difficulty in pulling all paediatric concepts together and collaborating with other faculty members to effectively convey the paediatric content [20]. In the Australian program accreditation guidelines published by the APC [2] the key areas which must be covered in an entry-level program include musculoskeletal, neurological, cardiorespiratory and electrophysical agents across all ages from acute to community contexts [2]. These guidelines, do not provide detailed guidance on the depth of content and skill development that is suitable for students to develop paediatric core competencies, potentially leaving graduates of some physiotherapy programs with a taste of paediatrics in each integrated subject without developing competencies relevant to paediatric clients in the particular clinical area. It is commonly understood by Australian physiotherapy program accreditation panels that there are not enough 5-week paediatric placement experiences for all physiotherapy students to have a paediatric placement experience assessed using the APP. Consequently, accreditation panels accept alternate models of practical experience compared to those in the clinical areas of neurological, musculoskeletal and cardiorespiratory physiotherapy which are typically undertaken in adult-oriented settings. The lack of detailed curriculum guidelines in this difficult to source placement area (i.e. paediatrics) means that the quality control for paediatric competencies compared to the more adult-oriented competencies in Australian physiotherapy programs is reduced. Currently, the APC accreditation standard 3.3 - *The quality and quantity of clinical education is sufficient to produce a graduate competent to practise across the lifespan in a range of environments and settings,* appears to be left open to interpretation for paediatrics as there is no defined expectation for coursework curriculum or practical experience. Whilst the authors of this paper are not suggesting that the APC mandate a set number of clinical placement hours or an exact syllabus, to ensure that programs are producing graduates competent to practise across the lifespan and to safeguard paediatric clients, further scrutiny in this area is warranted. It may be advantageous to have Australian guidelines available detailing minimum standards for paediatric curriculum and practical experience that physiotherapy programs could be benchmarked against by accreditation panels. A mandated bench-marking process for paediatric curriculum may also assist the universities to share and learn about new and innovative ways to develop paediatric specific skills and knowledge and this may be particularly helpful for programs who do not have paediatric physiotherapists on staff.

A stand-alone paediatric course was the second most common approach for curriculum delivery reported in our survey. This method of delivering paediatric content, if inclusive of case-based learning and laboratory experience, was recently recommended as the most effective method of teaching paediatric physiotherapy core competencies in a US based curriculum [25]. The desktop audit in the present study revealed that a stand-alone paediatric course (subject) was offered in only eight of the 20 universities audited. A potential reason for universities not having a stand-alone paediatric course could be due to limited time and prioritisation of paediatric content within the curriculum, which was reported as a barrier by multiple researchers in previous US-based literature [14–16, 19]. In the present study most participants recognised that offering a stand-alone paediatric course was a facilitator for adequately covering paediatric content. Universities and accreditation authorities could consider these findings when planning, developing and accrediting paediatric professional education to ensure that knowledge and skills relevant to a paediatric caseload (birth-18 years) are adequately covered and assessed.

The findings of this investigation are particularly important to consider with the Australian health-care model now introducing the NDIS funded care across the country. Historically, families of children requiring care for developmental or disability-related conditions were guided to apparent child-related services that employed suitably trained and or experienced paediatric physiotherapists with supervision and mentoring arrangements in situ. With the NDIS, the general population can now make an autonomous choice on the therapists they visit for their child’s disability support. The providers they choose to visit (either NDIS registered or private practitioner) may not necessarily have the level of skills and knowledge to treat a paediatric caseload (aged over 7 years). Additionally, physiotherapists servicing children may not have a supervision framework in place, as is commonly the case in publicly funded paediatric facilities (e.g. Hospitals and community health centres). This poses the question as to whether entry-level physiotherapists should be able to provide care to paediatric clients, without further training or supervision.

The SoP, APTA during the US education summit in 2012, identified five core competencies and endorsed a short list of common paediatric conditions for inclusion in US entry-level physiotherapy programs where evidence existed for physiotherapy interventions and these included; ASD, brachial plexus injury, Cerebral Palsy, congenital limb deficiencies, Cystic Fibrosis, DCD, developmental delay, Down Syndrome, Muscular Dystrophy, Myelomeningocele +/− with or without hydrocephalus and Torticollis +/− Plagiocephaly [20]. The results from the present survey demonstrated that Australian paediatric academic educators also perceived these conditions to be important for inclusion in entry-level physiotherapy curriculum. Additionally, three of the five core competency areas including human development, age-appropriate client management and family-centred care were considered important elements for inclusion by Australian academic educators. The final two core competency areas in the SoP, APTA curriculum guidelines [20] were not included in the present study and require further investigation in Australian contexts. The results of the present study have further validated the collective findings from previous US literature on this topic [9, 16, 20, 26, 27] and have contextualised these results to be relevant to the Australian higher education sector and physiotherapy profession. Consequently, the findings from this study could be used as a resource for Australian physiotherapy programs when developing or teaching paediatric physiotherapy content.

### Secondary findings

The Mann-Whitney U test revealed some significant differences between bachelor and master level programs for four paediatric conditions surveyed, but after conducting sensitivity analysis on the same results the differences were no longer apparent. The only difference observed was that masters level programs perceived growth-related injuries and sports and overuse injuries as slightly more important to cover in a program. As this difference was only observed in two out of the 31 conditions surveyed, it could be stated that most programs perceive it important to cover the documented paediatric conditions despite the level of the program (i.e. bachelor and masters). Our study also found that most of the paediatric content was delivered through lectures and tutorials and was assessed using written exams and quizzes. This suggests that a gap may exist in the practical assessment of paediatric competencies for physiotherapy students and this is likely due to the limited number of paediatric placement opportunities available in Australia. This was evidenced in addressing our fourth study aim, where a lack of paediatric-specific placements was noted as a barrier to many program’s paediatric curriculum. Half of the participants in the present study reported that they provided all students the opportunity to attend clinical placement in the field of paediatrics, however the length of time or expected learning outcomes from the placement (e.g. observation verses competencies) is unknown. If universities were mandated to benchmark their paediatric curriculum, universities who are underrepresented in paediatric placements, would have the opportunity to see how some universities manage to place all students in paediatric clinical experiences and assess their competencies for working safely and effectively with children. With the NDIS now active across Australia, it may be appropriate to consider new opportunities for paediatric placements, outside of hospital settings. Supervised visits to childcare and school environments, paediatric placements with private practices working with NDIS funded clients, university interprofessional paediatric clinics, simulated learning, in addition to the more commonly utilised community health and hospital environments, should all be considered as rich learning environments where physiotherapy students could develop paediatric specific competencies. Additionally, universities may need to look beyond traditional 5-week full time 1:1 supervised placement blocks, yet still offer adequate time and supervision for students to develop safe and effective skills that will serve children, infants and their families well.

Previous, research suggests that hands on experience assists in consolidating information and skills learnt at university [17]. Without all students undertaking placement experiences with paediatric clients, due to the identified lack of clinical placement availabilities, it is possible that only some students are being adequately prepared to manage a paediatric caseload as an entry-level physiotherapist. The lack of paediatric placement experiences for some students is likely to continue with growing cohort numbers in physiotherapy programs across Australia. Furthermore, in Australia, students attending placement types such as musculoskeletal, cardiorespiratory and neurological physiotherapy are consistently being examined by physiotherapists external to the university using the Assessment of Physiotherapy Practice (APP) [13] to ensure adequate knowledge and practical skills are being attained. Without all students undertaking a paediatric clinical placement assessed using the APP or another appropriate assessment tool, there is a risk that some physiotherapy students may graduate without assurances that they possess the skills, knowledge and attitudes to safely and effectively work with children. When placements are unable to be achieved, universities need to develop innovative and appropriate learning opportunities, including practical experiences within their curriculum to ensure that all students learn skills relevant to becoming safe, effective and efficient to work with paediatric populations and their families.

Limited financial resources were an identified barrier to the implementation of paediatric curriculum in our study and this barrier was also noted in previously published US-based literature [16, 19]. If universities who do not have permanent faculty members trained in paediatrics are indicating financial barriers, then it is possible that the same financial barriers may prohibit ‘buy-in’ of sessional paediatric staff to appropriately teach and assess the curriculum, bringing into question the quality and appropriateness of the paediatric content delivered and raises concerns that paediatric content may not be adequately included in program curriculum due to unavailability of qualified faculty. A stand-alone paediatric subject would potentially increase the likelihood of a paediatric physiotherapist being employed as a permanent staff member rather than short-term buy-in of staff occurring. Furthermore, if programs do not have staff appropriately trained to teach and assess paediatric content and skills, it is possible that this area of curriculum would become a low priority and potentially become diluted or even lost from subjects that are taught by physiotherapists with adult-based knowledge and skills only. Moreover, if a qualified faculty member with paediatric skills is employed on staff, development and quality control of a paediatric curriculum across the program and/or across a designated subject may be more likely to occur, as sessional staff members are unlikely to request information regarding the detailed scope of paediatric curriculum in a program, unless they are employed to do so. Further research is warranted to explore this area.

### Clinical relevance of study findings

The findings of this study are relevant to the APC, Australian university programs, curriculum developers, placement providers, the physiotherapy profession and the wider public community since the findings from our study provides the first published insights into the current landscape of paediatric curriculum in entry-level physiotherapy programs in Australia. With the information provided from this study, the program accreditation bodies or paediatric education interest groups could use this information to assist with developing or support the development of a minimum standards guideline for paediatric curriculum requirements for which universities could be held accountable. Universities can also use these study findings to compare and identify gaps within their curriculum and consider opportunities for benchmarking and ways to make appropriate changes to their program syllabus and staff resourcing. In supporting universities to produce safe entry-level physiotherapists, this research may help to increase the awareness of placement providers, that universities see a lack of paediatric placements as a barrier to the implementation of paediatric content. Therefore, placement providers in the future could increase available placement offers for students to practice and consolidate their knowledge and skills relevant to working with paediatric populations. Additionally, placement providers could work with universities to consider innovative and appropriate models of practical experience that provide learning and assessment opportunities in paediatrics for all students in Australian physiotherapy programs.

The collective contribution of accreditation bodies (e.g. APC), university representatives and paediatric clinicians, is needed to develop a minimum standard curriculum and assessment guideline, aimed at enhancing the skills and knowledge of entry-level physiotherapists for safe and effective practice with paediatric populations. If this can be achieved, the physiotherapy profession will be better able to assure the public that Australian physiotherapy graduates are adequately skilled to work as first-contact practitioners with children and their families.

### Limitations of this study

A limitation of this study relates to having a small sample size, due to the limited number of universities offering entry-level physiotherapy programs within Australia compared to larger countries such as the US. Non-responders may have been discouraged from completing this survey due to time commitments involved with participation. Another limitation was the potential bias within the results as lecturers invested in overlooking paediatric curriculum were the only participants requested to complete the survey from each university. As most of our participants were paediatric physiotherapists their perception of importance for covering paediatric content may have been inflated to favour inclusion of more paediatric content within a curriculum compared to non-paediatric staff. For this reason, the future research in this area should involve a wider representation of clinicians and potentially stakeholders such as the public community. When using the Likert scale to investigate the content covered and its perceived importance, no clear definition or classification of the scoring system was provided. This may have resulted in a subjective interpretation of the scale and may have decreased the accuracy of the results, as documented in previous published literature [28]. An additional limitation to this study was the design of the question regarding the use of paediatric placements to assess paediatric learning outcomes for students in their program. Almost half of respondents stated that a clinical placement was used to assess paediatric learning outcomes of all students in their program, however, national APP data does not suggest that there are adequate 5-week paediatric placements to validate these findings [29] suggesting that the placements may be atypical in length of time. The design of our survey limited the ability to determine if these placements were i) externally assessed using the APP or an alternate tool and ii) if the placement occurred over a full-time five-week duration or a shorter timeframe.

At the time of the analysis for this study, there were no responses providing further validation of the content in the desktop audits therefore the desktop audit data was analysed with publicly sourced information only. This is likely to be related to the increased time commitment to provide further detailed information, however there is a possibility that further paediatric content is being delivered and assessed that is not represented in the published subject outlines and therefore not represented in our desktop audit findings.

### Suggestions for future research

As this research was the first of its kind in Australia, the paediatric physiotherapy profession could build on this preliminary study with future investigations focusing on carrying out a similar survey with paediatric physiotherapy clinicians working within hospitals and the community. The findings from future research could complement the findings of the present study and help consolidate curriculum topics considered important for coverage in university programs. The wider physiotherapy profession and the public could also be surveyed to identify their perspective on the level of knowledge an entry-level physiotherapist has or should have to work with paediatric populations and such information could complement the findings of the present survey to contribute towards the development of minimum standards for paediatric curriculum. Additionally, future research should investigate the details of paediatric assessment in clinical practice to explore if paediatric competencies are being assessed in a valid manner after completion of clinical placement experiences. This may assist with ensuring graduates from Australian programs are safe, effective and efficient to work therapeutically with children and their families. Our study together with future complementary research could provide findings that may assist with developing minimum standards for paediatric curriculum and assessment in entry-level physiotherapy programs and consequently better assure the public of the knowledge, skills and attributes that all physiotherapists possess relevant to working with paediatric populations after graduating from an Australian physiotherapy program. To develop minimum standards for Australian entry-level physiotherapy paediatric curriculum (including clinical experiences) the recommended research above, must first be undertaken to fully understand the expectation of key stakeholders regarding paediatric physiotherapy curriculum. With this information, paediatric teaching leads may then collaborate through targeted workshops or Delphi style consensus methods [30] to achieve consensus on what should be included in Australian minimum standards for pediatric physiotherapy entry-level curriculum.

## Conclusion

As no documented standard or core competencies exist for paediatric curriculum in Australia there is extensive variability amongst Australian entry-level programs for preparing physiotherapists to work safely and effectively with children. The results of this study suggest there are commonly agreed imperative paediatric curriculum content areas to be covered in training entry-level physiotherapists. Most universities surveyed stated the perceived importance of many paediatric content areas to higher than the level it was being covered in their program. The main barriers to the implementation of paediatric curriculum included; crowded curriculum, limited financial resources and inadequate paediatric placements. Findings from this study may help inform the development of minimum standards for paediatric-specific knowledge, skills and attributes for students in entry-level physiotherapy curriculum across Australia.

## Additional file

Additional file 1: Copy of Survey titled Paediatric Physiotherapy Curriculum: An audit of Australian physiotherapy entry-level programs. (PDF 1496 kb)

## Abbreviations

AHPRA: Australian Health Practitioner Regulation Agency
APC: Australian Physiotherapy Council
APP: Assessment of Physiotherapy Practice
ECEI: Early Childhood Early Intervention
IPE: Interprofessional Health Education
NDIS: National Disability Insurance Scheme
OSCE: Objective Structured Clinical Exam
SD: Standard Deviation
US: United States of America
