# Supplementary material for: Paediatric Physiotherapy curriculum: an audit and survey of Australian entry-level Physiotherapy programs
Source: BMC Med Educ. 2019 Apr 16;19:109. doi: 10.1186/s12909-019-1540-z (PMC6469150; doi:10.1186/s12909-019-1540-z)

Paediatric Physiotherapy Curriculum: An audit of Australian physiotherapy entry-level programs

Participant Information Sheet

PLEASE READ THIS CAREFULLY BEFORE PROCEEDING

Ethics Reference Number: 16162

My name is Dr Nikki Milne and I am currently completing a research study titled Paediatric physiotherapy curriculum: An audit of Australian physiotherapy entry-level programs with two Doctor of Physiotherapy students from Bond University: Karen Mistry and Emi Yonezawa.

The focus of our survey is on physiotherapy entry-level programs. Contact information of physiotherapists who teach into your program were sourced from university websites.

What is involved in this research project?

As a part of this study, we will invite you to complete an online consent form, staff profile and survey through Survey Monkey. A desktop audit of your university paediatric physiotherapy curriculum has been completed and will be provided to you after completion of this survey to review and edit if needed. A follow-up phone or Skype interview may be conducted to clarify any further information and answer any questions.

The following table provides some information about the online forms we plan to use in this study and your time commitment should you consent to participate in the study.

Tool	What you can expect	
Step 2 – Consent and staff profile form	The purpose of this step is to gain consent by the physiotherapist lecturer who teaches within the paediatric curriculum to participate in the study and to outline their preferred contact details. It will take approximately **10 minutes** to complete this step 2.	This form is specific to the physiotherapy lecturers
Step 3 - Survey	The online survey will be completed through Survey Monkey. The first component of the survey will ask the physiotherapy lecturer about their role at the university such as their current position, hours worked and years of experience within the field. The second component of the survey will consist of open and closed questions, which will further explore what paediatric content is covered within their curriculum, to identify if there are any gaps within the content, what components of paediatric physiotherapy should be included as an essential part of entry-level physiotherapy programs and to recognise any barrier or facilitators of the inclusion and delivery of the paediatric curriculum.<br><br>It will take you approximately **30 minutes** to complete this online survey, which can be saved and resumed at a later time. You will only need to complete it once. No persons other than the research team will be able to access the data within your profile (unless you choose to provide this information to others).	This survey is specific to the research study.
Step 4: Review and edit desktop audit	A desktop audit of each university's course curriculum will be developed and given to each participant to review and edit. This step will take approximately **30 minutes** to complete in a Word document emailed to each participant.	This audit is specific to the university's curriculum
Step 5: Follow-up call or Skype Interview	After the submission of the online survey and review/edit of the desktop audit has been analysed, you may be contacted with a follow-up phone or Skype interview to clarify any information provided. A meeting time will can be organised to suit the participant. If contacted it will take approximately **15-20min** to further clarify any information.	

Who can participate in this research project?

We are seeking physiotherapist lecturing on paediatric curriculum into entry-level physiotherapy programs in Australia. Participation in this study is **completely voluntary** and you may withdraw at any time without risking any negative consequences.

What are the potential risks and benefits of this research project?

There are no **perceived risks** associated with you participating in this study. All data will be made non-identifiable prior to publication. There will be no risk of universities or participants being identified through their survey results or desktop audit. To prevent this, only the research team (Dr. Nikki Milne and students Karen Mistry and Emi Yonezawa) will be able to identify responses and will only deliver the collaborated data in a non-identifiable format for the draft and published manuscript. Should any participants become distressed about their staff profile, survey results or desktop audit after submission, they will have the opportunity to discuss with the research team via phone or Skype meeting regarding any concerns they may have, which will remain confidential.

There are a number of **potential benefits** for physiotherapy lecturers and universities participating in this study. The analysed data after publication will be made available to all participants to compare their university's curriculum against a developed benchmark of curriculum at universities in Australian who participated in the study. The information provided to physiotherapy lecturers teaching into the paediatric curriculum and universities may be utilised to further develop their curriculum whist comparing it to other Australian physiotherapy programs. In addition, universities and physiotherapy lecturers can use this information to demonstrate that their physiotherapy program contains an approved level of paediatric curriculum to gain APC and TEQSA approval. Finally, all participants will have the opportunity to have a follow-up phone or Skype meeting to clarify responses and to discuss any further questions by participants.

What is my responsibility as a participant in this research project?

To actively participate in the project by completing the forms listed in the table above.

How will my data results be used?

All the data collected in this study will be treated with **complete confidentiality** and not made accessible to any person outside of the research team for this project without your permission. Data will be stored in a **secured location** at Bond University for a period of five years in accordance with the guidelines set out by the Bond University Human Research Ethics Committee. The consent form, staff profile and survey will be completed through Survey Monkey and will only be accessible by the research team who will have a username and password to gain access to the information. The desktop audit will be completed through a Word document, which will be stored on a private hard drive kept by the research team. At all times the information provided will remain confidential and will be made non-identifiable prior to publication.

What is the significance of this research project?

The proposed study is a new and novel area of research to be undertaken at Bond University, which is relevant

If you have any enquires regarding this research project or experience distress from participation in this research, please contact any of the researchers whose details are listed below.

We thank you for taking the time to assist us with this research.

Yours sincerely,

Assistant Professor Dr Nikki Milne
PhD, MPhty, BExSc, BEd, MEd (ECE), Grad Cert Clinical Ed
Tel: +61 7 5595 4155
Email: nmilne@bond.edu.au

Doctor of Physiotherapy student Karen Mistry
Tel: +61 449 158 751
Email: karen.mistry@student.bond.edu.au

Doctor of Physiotherapy student Emi Yonezawa
Tel: +61 430 332 180
Email: emi.yonezawa@student.bond.edu.au

Should you have any complaints concerning the manner in which this research is being conducted, please make contact with:
Bond University Human Research Ethics Committee,
Bond University Office of Research Services,
Bond University, Gold Coast, 4229
Tel: +61 7 5595 4194 Fax: +61 7 5595 1120
Email: buhrec@bond.edu.au

Consent form - Participant

Ethics Reference Number: 16162

Voluntary Consent - Participant

By providing my consent below, I agree to take part in the Bond University research project, which aims to explore the status (content and assessment) of paediatric physiotherapy knowledge and skills in entry-level programs in Australia as described on the Participant Information Sheet for the study titled: Paediatric physiotherapy curriculum: An audit of Australian physiotherapy entry-level programs.

1. **I have read the Participant Information Sheet and Participant Informed Consent Form and clearly understand the content and what is being asked of me as a participant in the study.**
2. **I clearly understand the potential risks and benefits of my participation in this study.**
3. **I have had the opportunity to ask questions about the study and the questions I have asked have been answered to my satisfaction. I also understand that I can ask questions about the study and my participation at any time.**
4. **I understand that the data I provide will be handled in a confidential manner and that any publication of the data I provide will be de-identified and included collectively with the data of other institutions of higher education, as percentages, means, frequencies and ranges. Additionally, open-ended questions will be thematically analysed and key themes presented.**
5. **I understand that my participation is entirely voluntary and that I can withdraw from the study at any time without penalty or consequence.**
6. **I understand that my staff profile, surveyresults and desktop audit review/edit will not be shared with any persons outside of the research team except through non-identifiable research publications.**

* 1. I give my consent to participate in the study titled: Paediatric physiotherapy curriculum: An audit of Australian physiotherapy entry-level programs.

- ◯ Yes
- ◯ No, please provide a reason

Participant Information

2. Please enter your name.

3. What is your gender?

- Female
- Male
- Undisclosed

* 4. Please enter the name of the University you are currently employed at.

* 5. Please provide your best contact details

Phone

Email

Survey Introduction

With physiotherapists being recognised as first contact practitioners in Australia, it is expected that all graduates from entry-level programs will have a foundation of knowledge and skills to adequately assess and treat paediatric clients. The aim of this survey is to explore the status of paediatric physiotherapy knowledge and skills delivered in entry-level physiotherapy programs in Australia. The survey has been structured into 6 key themes: demographic of paediatric teaching staff, typical development, paediatric diagnosis (atypical development), paediatric examination/assessment, paediatric intervention and curriculum. In addition, this survey will provide the opportunity to explore barriers and facilitators (including paediatric academic workforce) to the delivery of paediatric physiotherapy curriculum and how standards for core paediatric curriculum can be developed.

Throughout this survey the following definition of syllabus and curriculum have been used:

- **A *syllabus* is an outline or summary of the subjects to be covered in a course.(Collins, 2017)**
- **A *curriculum* is all the different courses of study that are taught in a school, college, or university. (Collins, 2017)**

The following survey will take approximately 30 minutes to complete. It can be saved and resumed later if needed.

Please answer the following questions in relation to the entire paediatric curriculum across your program.

Theme I: Paediatric Teaching Staff Demographic / Profile

* 6. What is your highest earned academic degree? (Please select one or all as they apply)

- [ ] Bachelor of Physiotherapy
- [ ] Bachelor of Physiotherapy with Honours
- [ ] Master of Physiotherapy
- [ ] Master of Physiotherapy with Honours
- [ ] Other (please specify)
- [ ] Doctor of Physiotherapy (Masters extended)
- [ ] Diploma of Physiotherapy
- [ ] Post-graduate Master of Physiotherapy (Paediatrics)
- [ ] PhD

7. In what country did you undertake your entry-level (physiotherapy) academic degree?

8. What was the name of your entry-level degree to become a physiotherapist?

9. What was the name of the institution where you completed your entry-level physiotherapy degree?

10. What other formal qualifications do you hold?

11. How many years have you been a physiotherapist?

12. Are you currently enrolled in any post-graduate programs?

- No
- Yes, please state:

13. Have you undertaken clinical specialty post-graduate training in any of the following areas?

- ☐ Cardiopulmonary
- ☐ Paediatrics
- ☐ Musculoskeletal
- ☐ Sports
- ☐ Neurological
- ☐ Gender Health
- ☐ Neurodevelopmental therapy
- ☐ Education
- ☐ Other (please specify)

14. What is your current position within your faculty?

- ○ Full time
- ○ Adjunct
- ○ Part time
- ○ Sessional/Contract
- ○ Other (please specify)

Theme I: Paediatric Teaching Staff Demographic / Profile

15. If not full-time, what is your Full Time Equivalent (FTE)?

[ ]

16. How many years have you been teaching into the paediatric curriculum in your current position?

[ ]

17. Have you been involved in teaching into paediatric curriculum in other institutions?

- ◯ No
- ◯ Yes, how long

[ ]

18. What is your current academic title?

- ◯ Teaching Fellow
- ◯ Senior Teaching Fellow
- ◯ Lecturer
- ◯ Senior Lecturer
- ◯ Assistant Professor
- ◯ Associate Professor
- ◯ Professor
- ◯ Adjunct
- ◯ Sessional Teaching Staff
- ◯ Other (please specify)

[ ]

Current Level of Coverage in Curriculum

19. **DOES THE SYLLABUS INCLUDE** content regarding the typical progression of a child as they develop and grow older by acquiring and refining knowledge, behaviors, and skills?

Please select the most appropriate response for the following. My program syllabus aims to ensure that students:

	Not at all	Not very well	Somewhat	Well	Very Well
Develop foundation knowledge of prenatal development and birth	○	○	○	○	○
Develop foundation knowledge of the theories of childhood development and learning	○	○	○	○	○
Demonstrate knowledge of developmental motor milestones	○	○	○	○	○
Understand the importance of therapeutic play within diverse family, cultural, community and societal contexts	○	○	○	○	○
Understand when a child should provide assent and appropriately gaining parent / carer consent	○	○	○	○	○
Demonstrate knowledge of developmental milestones in the social-emotional and speech and language domains	○	○	○	○	○

Other (please specify)

Theme II: Knowledge of Typical Development

Perceived Importance in Paediatric Curriculum

20. **HOW IMPORTANT DO YOU THINK** it is to cover content which aims to ensure that student's:

Please select the most appropriate response for the following:

	Strongly Disagree	Disagree	Neutral	Agree	Strongly Agree
Develop foundation knowledge of prenatal development and birth	◯	◯	◯	◯	◯
Develop foundation knowledge of the theories of childhood development and learning	◯	◯	◯	◯	◯
Demonstrate knowledge of developmental motor milestones	◯	◯	◯	◯	◯
Understand the importance of therapeutic play within diverse family, cultural, community and societal contexts	◯	◯	◯	◯	◯
Understand when a child should provide assent and appropriately gaining parent / carer consent	◯	◯	◯	◯	◯
Demonstrate knowledge of developmental milestones in the social-emotional and speech and language domains	◯	◯	◯	◯	◯

Current Level of Coverage in Curriculum

21. DOES THE SYLLABUS INCLUDE the aetiology, pathophysiology, diagnosis, signs and symptoms, prognosis and management of the following diseases, disorders and conditions in paediatric populations?

Please select the most appropriate response for the following:

Cardiorespiratory and cardiovascular

	Not at All	Not Very Well	Somewhat	Well	Very Well
Asthma	○	○	○	○	○
Cardiomyopathies	○	○	○	○	○
Congenital Heart Defect	○	○	○	○	○
Cystic Fibrosis	○	○	○	○	○

Other (please specify)

22. DOES THE SYLLABUS INCLUDE the aetiology, pathophysiology, diagnosis, signs and symptoms, prognosis and management of the following diseases, disorders and conditions in paediatric populations?

Please select the most appropriate response for the following:

Musculoskeletal

	Not at All	Not Very Well	Somewhat	Well	Very Well
Amputation	○	○	○	○	○
Congenital Conditions (e.g. foot deformities, contractures, hip dysplasia, limb deficiencies)	○	○	○	○	○
Growth-related injuries	○	○	○	○	○
Juvenile rheumatoid arthritis	○	○	○	○	○
Muscular Dystrophy	○	○	○	○	○
Osteogenesis Imperfecta	○	○	○	○	○
Other rheumatic and connective tissue diseases	○	○	○	○	○
Scoliosis	○	○	○	○	○
Sports and Overuse Injuries	○	○	○	○	○
Torticollis	○	○	○	○	○
Plagiocephaly	○	○	○	○	○

Other (please specify)

23. DOES THE SYLLABUS INCLUDE the aetiology, pathophysiology, diagnosis, signs and symptoms, prognosis and management of the following diseases, disorders and conditions in paediatric populations?

Please select the most appropriate response for the following:

Neurological

	Not at All	Not Very Well	Somewhat	Well	Very Well
Brachial plexus birth injuries	○	○	○	○	○
Cerebral palsy	○	○	○	○	○
Cognitive impairment and pervasive developmental delay	○	○	○	○	○
Complications in premature infants	○	○	○	○	○
Neurodevelopmental conditions (e.g. specific learning disorder, ADHD, DCD, ASD)	○	○	○	○	○
Genetic disorders/ chromosomal disorder (e.g. Down syndrome)	○	○	○	○	○
Infectious diseases	○	○	○	○	○
Myelodysplasia and Hydrocephalus	○	○	○	○	○
Sensory motor impairments	○	○	○	○	○
Spina Bifida	○	○	○	○	○
Traumatic brain injury and Spinal Cord Injury	○	○	○	○	○

Other (please specify)

24. DOES THE SYLLABUS INCLUDE the aetiology, pathophysiology, diagnosis, signs and symptoms, prognosis and management of the following diseases, disorders and conditions in paediatric populations?

Please select the most appropriate response for the following:

Other

	Not at All	Not Very Well	Somewhat	Well	Very Well
Burns	○	○	○	○	○
Cancers	○	○	○	○	○
Drug exposure/ Lead poisoning	○	○	○	○	○
Failure to thrive	○	○	○	○	○
Immune deficiency syndrome	○	○	○	○	○

Other (please specify)

Perceived Importance in Paediatric Curriculum

25. HOW IMPORTANT DO YOU THINK is it to include the aetiology, pathophysiology, diagnosis, signs and symptoms, prognosis and medical management of the following diseases, disorders and conditions?

Please select the most appropriate response for the following:

Cardiorespiratory and cardiovascular

	Strongly Disagree	Disagree	Neutral	Agree	Strongly Agree
Asthma	○	○	○	○	○
Cardiomyopathies	○	○	○	○	○
Congenital Heart Defect	○	○	○	○	○
Cystic Fibrosis	○	○	○	○	○

26. HOW IMPORTANT DO YOU THINK is it to include the aetiology, pathophysiology, diagnosis, signs and symptoms, prognosis and medical management of the following diseases, disorders and conditions?

Please select the most appropriate response for the following:

Musculoskeletal

	Strongly Disagree	Disagree	Neutral	Agree	Strongly Agree
Amputation	○	○	○	○	○
Congenital Conditions (e.g. foot deformities, contractures, hip dysplasia, limb deficiencies)	○	○	○	○	○
Growth-related injuries	○	○	○	○	○
Juvenile rheumatoid arthritis	○	○	○	○	○
Muscular Dystrophy	○	○	○	○	○
Osteogenesis Imperfecta	○	○	○	○	○
Other rheumatic and connective tissue diseases	○	○	○	○	○
Scoliosis	○	○	○	○	○
Sports and Overuse Injuries	○	○	○	○	○
Torticollis	○	○	○	○	○
Plagiocephaly	○	○	○	○	○

27. HOW IMPORTANT DO YOU THINK is it to include the aetiology, pathophysiology, diagnosis, signs and symptoms, prognosis and medical management of the following diseases, disorders and conditions?

Please select the most appropriate response for the following:

Neurological

	Strongly Disagree	Disagree	Neutral	Agree	Strongly Agree
Brachial plexus birth injuries	○	○	○	○	○
Cerebral palsy	○	○	○	○	○
Cognitive impairment and pervasive developmental delay	○	○	○	○	○
Complications in premature infants	○	○	○	○	○
Neurodevelopmental conditions (e.g. specific learning disorder, ADHD, DCD, ASD)	○	○	○	○	○
Genetic disorders/ chromosomal disorder (e.g. Down syndrome)	○	○	○	○	○
Infectious diseases	○	○	○	○	○
Myelodysplasia and Hydrocephalus	○	○	○	○	○
Sensory motor impairments	○	○	○	○	○
Spina Bifida	○	○	○	○	○
Traumatic brain injury and Spinal Cord Injury	○	○	○	○	○

28. HOW IMPORTANT DO YOU THINK is it to include the aetiology, pathophysiology, diagnosis, signs and symptoms, prognosis and medical management of the following diseases, disorders and conditions?

Please select the most appropriate response for the following:

Other

	Strongly Disagree	Disagree	Neutral	Agree	Strongly Agree
Burns	○	○	○	○	○
Cancers	○	○	○	○	○
Drug exposure/ Lead poisoning	○	○	○	○	○
Failure to thrive	○	○	○	○	○
Immune deficiency syndrome	○	○	○	○	○

Current Level of Coverage in Curriculum

29. DOES THE SYLLABUS INCLUDE the reasoning of the physiotherapy assessment process, involving purpose, method, analysis and planning within the paediatric population?

Please select the most appropriate response for the following:

	Not at All	Not Very Well	Somewhat	Well	Very Well
Client / Parent Interview	○	○	○	○	○
Physical Assessment	○	○	○	○	○
Ergonomics and body mechanics	○	○	○	○	○
Gait, locomotion and balance	○	○	○	○	○
Joint integrity and mobility	○	○	○	○	○
Motor control and motor learning	○	○	○	○	○
Motor skills	○	○	○	○	○
Muscle performance (strength, power, endurance)	○	○	○	○	○
Neuromotor and sensory assessment (development and integration)	○	○	○	○	○
Orthotic, protective and supportive devices	○	○	○	○	○
Posture	○	○	○	○	○
Range of motion (muscle length)	○	○	○	○	○
Reflex integrity	○	○	○	○	○
Ventilation and respiration/ Gas exchange	○	○	○	○	○
Cardiorespiratory fitness	○	○	○	○	○

	Not at All	Not Very Well	Somewhat	Well	Very Well
Outcome measures (e.g. AIMS, NSMDA, GMFCS, BOT2, MABC2, TGMD2 etc.)	◯	◯	◯	◯	◯
Clinical reasoning tools (e.g. ICF)	◯	◯	◯	◯	◯
Understanding the role of the members of the interprofessional paediatric team	◯	◯	◯	◯	◯

Other clinical reasoning tools used (please specify)

Perceived Importance in Paediatric Curriculum

30. HOW IMPORTANT DO YOU THINK is it to include the reasoning of the physiotherapy assessment process, involving purpose, method, analysis and planning within the paediatric population? Please select the most appropriate response for the following:

	Strongly Disagree	Disagree	Neutral	Agree	Strongly Agree
Client / Parent Interview	○	○	○	○	○
Physical Assessment	○	○	○	○	○
Ergonomics and body mechanics	○	○	○	○	○
Gait, locomotion and balance	○	○	○	○	○
Joint integrity and mobility	○	○	○	○	○
Motor control and motor learning	○	○	○	○	○
Motor skills	○	○	○	○	○
Muscle performance (strength, power, endurance)	○	○	○	○	○
Neuromotor and sensory assessment (development and integration)	○	○	○	○	○
Orthotic, protective and supportive devices	○	○	○	○	○
Posture	○	○	○	○	○
Range of motion (muscle length)	○	○	○	○	○
Reflex integrity	○	○	○	○	○
Ventilation and respiration/ Gas exchange	○	○	○	○	○
Cardiorespiratory fitness	○	○	○	○	○
Outcome measures (e.g. AIMS, NSMDA, GMFCS, BOT2, MABC2, TGMD2 etc.)	○	○	○	○	○
Clinical reasoning tools (e.g. ICF)	○	○	○	○	○
Understanding the role of the members of the interprofessional paediatric team	○	○	○	○	○

Current Level of Coverage in Curriculum

31. DOES THE SYLLABUS INCLUDE a foundation of content regarding different approaches to physiotherapy intervention and tools to undertake the treatment of paediatric clients?

Please select the most appropriate response for the following in relation to the paediatric curriculum:

	Not at All	Not Very Well	Somewhat	Well	Very Well
Manual therapy or Positioning and handling	○	○	○	○	○
Family/patient-centered care	○	○	○	○	○
Therapeutic exercises	○	○	○	○	○
Play-based exercises	○	○	○	○	○
Functional training in self-care and in-home management	○	○	○	○	○
Functional training for use in school or play, in the community and leisure integration / reintegration	○	○	○	○	○
Prescription and application of equipment and devices (assistive devices, orthotics, prosthetics)	○	○	○	○	○
Airway clearance techniques	○	○	○	○	○
Electrotherapeutic and mechanical modalities	○	○	○	○	○
Behaviour management	○	○	○	○	○
Understanding the role of the members of the interprofessional paediatric team	○	○	○	○	○
Use of goal setting for treatment planning	○	○	○	○	○

Other (please specify)

Perceived Importance in Paediatric Curriculum

32. HOW IMPORTANT DO YOU THINK is it to include a foundation of content regarding different approaches to physiotherapy intervention and tools to undertake the treatment of paediatric clients?

Please select the most appropriate response for the following in relation to the paediatric curriculum:

	Strongly Disagree	Disagree	Neutral	Agree	Strongly Agree
Manual therapy or Positioning and handling	○	○	○	○	○
Family/patient-centered care	○	○	○	○	○
Therapeutic exercises	○	○	○	○	○
Play-based exercises	○	○	○	○	○
Functional training in self-care and in-home management	○	○	○	○	○
Functional training for use in school or play, in the community and leisure integration / reintegration	○	○	○	○	○
Prescription and application of equipment and devices (assistive devices, orthotics, prosthetics)	○	○	○	○	○
Airway clearance techniques	○	○	○	○	○
Electrotherapeutic and mechanical modalities	○	○	○	○	○
Behaviour management	○	○	○	○	○
Understanding the role of the members of the interprofessional paediatric team	○	○	○	○	○
Use of goal setting for treatment planning	○	○	○	○	○

Theme VI: Curriculum

33. How is the paediatric content assessed within the curriculum?
Select from the options below (more than one answer is accepted)

- [ ] Quizzes
- [ ] Practical exam (OSCE)
- [ ] Written Exam
- [ ] Clinical Placement for all Students
- [ ] Seminar / oral presentation
- [ ] Other (please specify)

34. How is the paediatric content delivered to students?
Select from the options below (more than one answer is accepted)

- [ ] Lectures
- [ ] Workshops
- [ ] Tutorials
- [ ] Clinical Placement
- [ ] Problem based learning classes
- [ ] Simulated Learning
- [ ] Independent study
- [ ] Flip Classes
- [ ] Online modules
- [ ] Other (please specify)

35. How is the paediatric syllabus taught throughout the curriculum?

- [ ] Across subjects as a lifespan approach
- [ ] A stand alone paediatric subject within the curriculum
- [ ] Other (please specify)

36. In your point of view what are your perceived strengths of your curriculum regarding paediatric content?

37. In your point of view, what are the perceived weaknesses of the curriculum regarding paediatric content?

NOTE: Please remain aware that no individual university will be identified in ANY publication of the findings for this survey.

38. Are there any areas of paediatric content/skills that you currently do not cover in your program that you feel should be covered to adequately prepare a student for entry-level practice?

39. Within your institution are any of the following barriers to the implementation and development of **curriculum** in the field of**paediatric** physiotherapy? Please select yes or no for all the questions below as seen fit

	Yes	No
Limited number of practical or placement opportunities available in hospital/clinics, private practice, school and community based programs with children with special needs	○	○
Crowded curriculum due to the requirements of the professional practice guidelines provided by Australian Physiotherapy Council to be eligible for registration	○	○
Lack of qualified personnel available to teach within the educational field of paediatric physiotherapy curriculum	○	○
Organisational structure of the institution of higher education	○	○
Limited institutional, state or federal financial resources	○	○
Lack of coordination among states and institutions of higher education to develop a collaborative curriculum or standard of education to be taught within the curriculum	○	○
Lack of prioritisation of curriculum space for paediatric content	○	○

Other (please specify)

40. Please indicate what you believe to be **facilitators** to the implementation and development of **paediatric curriculum** in your program / institution.

41. Within your institution are any of the following barriers to the implementation and development of **interprofessional education** and training for students, in the field of**child health and development**?

Please select yes or no for all the questions below as seen fit

	Yes	No
Limited number of INTERPROFESSIONAL practical or placement opportunities available in hospital/clinics, private practice, school and community based programs with children with special needs	◯	◯
Crowded curriculum due to the requirements of the professional practice guidelines provided by Australian Physiotherapy council for licensing or certification	◯	◯
Lack of qualified personnel available to teach within the educational field of CHILD HEALTH AND DEVELOPMENT	◯	◯
Organisational structure of the institution of higher education	◯	◯
Limited institutional, state or federal financial resources	◯	◯
Lack of coordination among states and institutions of higher education to develop a collaborative curriculum or standard of education to be taught within the curriculum	◯	◯
Timetabling challenges	◯	◯
Space not suitable for inter-professional education	◯	◯

Other (please specify)

42. Please indicate what you believe to be **facilitators** to the implementation and development of **interprofessional education** and training for physiotherapy students in the field of child health and development in your program / institution.

43. Do you have any other comments that you would like to provide about paediatric physiotherapy curriculum in entry-level programs in Australia?

Thank You

Thank you for taking the time to participate in this survey

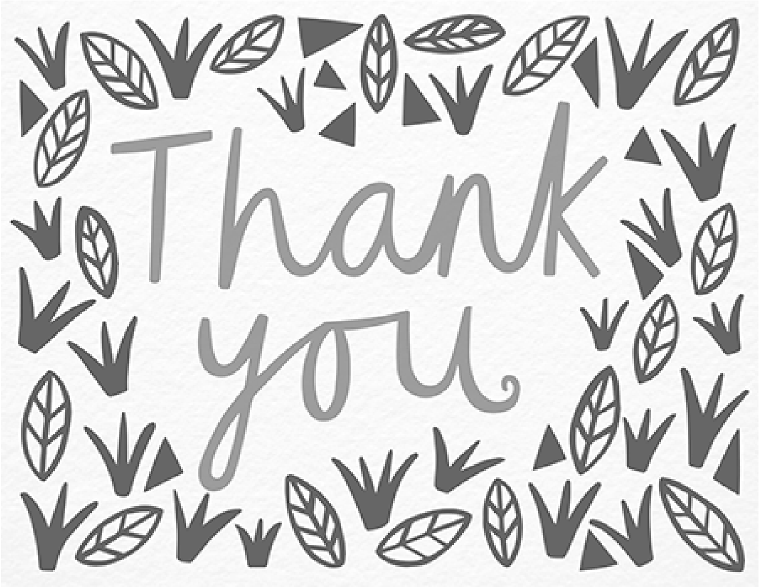

Supplement: Supplementary file 1 — Copy of Survey titled Paediatric Physiotherapy Curriculum: An audit of Australian physiotherapy entry-level programs. (PDF 1496 kb) [file 12909_2019_1540_MOESM1_ESM.pdf]
